# Southern Dental Association

**Published:** 1887-11

**Authors:** 


(TO BE CONTINUED.)

---

### SOUTHERN DENTAL ASSOCIATION.

### NINETEENTH ANNUAL SESSION AT OLD POINT COMFORT, VA., 1887.

REPORTED FOR THE INDEPENDENT PRACTITIONER, BY "MRS. M. W. J."

CONTINUED FROM PAGE 546.

### WEDNESDAY AFTERNOON SESSION.

The subject of Operative Dentistry was continued. Dr. Staples, of Sherman, Texas, read a paper, entitled "The Causes of Failures in Fillings." He attributed ninety-five per cent. of failures to lack of thoroughness in one way or another, beginning with the tutor in selecting the material out of which to make the future dentist. There is often not only "electro-chemical incompatibility between filling material and dentos," but also electro magnetic incompatibility between both the operator and his patient, and the operator and his work. The dentist is born, not made. If he is born a slip-shod man, there will be lack of thoroughness in all that he does. If he is born stingy, there will be lack of thoroughness from not properly cleansing out the cavity, for fear that it will be made too large, with the consequent demand for too much material. He concluded his paper by saying that we want thoroughness at every point; a thorough application of one end of the instrument, and a thorough dentist at the other end of it.

Dr. Parramore, of Hampton, Va., read a paper, entitled "Aseptic Sponge." He said that man was but an aggregation of cells in various stages of development, the white blood-corpuscles building up tissue, which is transformed into bone, muscle, teeth, hair, etc. Under the microscope we see in a single drop of deep sea water myriads of atoms quivering with life, but lacking the proper environment to develop into something higher. Immerse a log of wood or a piece of sponge in the water, and it is at once filled with these atoms, developing into new phases of life in obedience to their new environment. These atoms are prototypes of other bodies, which require similar protection and assistance to reach their highest development. This is illustrated in sponge-grafting, when the surgeon or the dentist steps in to aid nature in the work of repair. In inflammation there is an abnormal flow of blood to the parts and an increased supply of pabulum for repairs in tissue-building; the result depends on the ability of the parts to appropriate the pabulum. A pulp irritated by exposure makes an effort to protect itself; there is an increased flow of blood, with a deposition of lime-salts; we want the formation of secondary dentine—not of pulp-stones. If we place a small piece of aseptic sponge at the point of exposure, corpuscles will fasten themselves upon it, and, cemented with fibrin and nourished with pabulum, an adamantine barrier will be raised, behind which the pulp may rest secure.

Dr. Parramore stated that though his experience in this direction still lacked the final test of time, he felt well satisfied with the result of pulps capped in this manner six months ago, and hoped the matter would be tested by others. The sponge, after being thoroughly cleansed under microscopic examination, is sterilized in a one to five hundred solution of bi-chloride of mercury at a temperature of from 180° to 200° F. It is then dried without squeezing, and kept as free from contamination as possible. It is applied dry to the exposed pulp, and the cavity filled with oxy-phosphate, everything used in the operation, including the hands, being bathed in the bi-chloride solution. He also suggested the experimental application of a minute portion of aseptic sponge to the apex of root-canals, to induce the formation of bone material at that point.

Dr. Geo. H. Winckler, of Augusta, Ga., read a paper, entitled "Soft Gold Foils," claiming for soft gold the advantages of being worked very rapidly, thus saving the time of the operator and the strength of patients, especially women and children, the avoidance of the rubber-dam, so objectionable to many, its perfect adaptation to the walls of a cavity, protecting them against bruises. He commends soft foil, especially for all simple cavities with walls intact, for cavities under the free margins of the gums, in simple approximal cavities where knuckling is not required, or if to be knuckled, cohesive foil can be used for contouring, welding it into undercuts when the cavity is two-thirds filled with soft gold.

Dr. Winckler uses soft gold in pellets made by folding a square piece upon itself, making a mat which is crumpled and rolled in the fingers into an oblong pellet pointed at one end. He uses smooth instruments, carefully avoiding pushing through the mass, but compressing thoroughly. For crown cavities he uses forcep-pluggers, having a pad under the jaw for one beak and a condensing point on the other, sometimes utilizing the power of the muscles of the jaw by having the patient bite upon a pad of block tin soldered to the beaks of the forceps. While advocating the use of soft foil in the cases named, he combines both soft and cohesive gold in many instances, and has thus the advantages of both.

*Dr. W. H. Morgan*—Took issue with Dr. Marshall as to the superiority of new amalgams over the old, and with Dr. Staples as to the "born dentist," education making the man. Replying to Dr. Winckler, he said that the fact that the profession had so largely abandoned soft gold was proof that it was not the best.

*Dr. McKellops*—Replied that it was not dental education that made such men as John Hunter, and the fathers of dentistry.

*Dr. Staples*—Said that to educate meant to draw out, and education could not draw out of a man what was not in him. To be a good dentist he must be born to the vocation; in other words, he must have certain natural capacities.

*Dr. Storey*, of Dallas, Texas—Had never seen a tooth filled with soft gold that would not have been better filled with cohesive gold. He had not been successful in capping pulps, but he intended to try Dr. Parramore's plan.

*Dr. Beach*, of Clarksville, Tenn.—Thought the true middle ground was to acquire the greatest possible skill, not only in the use of both soft foil and cohesive gold, but also of the plastic materials, he who saves the greatest number of teeth being the best practitioner. The man who confines himself to one material deprives himself of many advantages, and loses many teeth that he might otherwise save.

Dr. J. B. Hodgkin, of the Baltimore College, read a paper, entitled "Amalgams." He said that the making and working of amalgams was based on occult, hidden laws of metallurgy, like causes *not* producing like effects in metallurgic combinations, for the behavior of an alloy could not be predicted from a knowledge of its constituents. After citing certain facts, as that two soft metals, as tin and lead, when combined, make an alloy harder than either of its constituents; that tin and copper, both soft, in combination become hard and tough, but not malleable; that tin (one) and copper (two) makes an alloy hard as steel and brittle as glass, while the addition of more copper and a very small proportion of phosphorus, itself soft and wax-like, gives a compound with greater tensile strength than steel; that iridium, which can only be drilled with the diamond or fused with the oxy-hydrogen blow-pipe, by the addition of a little phosphorus melts rapidly, etc., etc., showing that experiment only can solve the intricate problem of alloys, and only the most brilliant imagination follow out the varying molecular arrangements and chemical action of the various combinations of which metallic elements are susceptible. The addition of mercury induces a still more complex crystallization. The spheroidal tendency, creeping or changing shape, shrinkage on the one hand and expansion on the other, of these alloys, have not yet been effectually controlled, while time brings still other changes. The chemical action of these alloys on tooth-substance is still unsettled; those which turn dark seem to be the best tooth preservers. The chemistry of the nineteenth century is silent as to why this is so, though various theories have been advanced in regard to the action of the sulphides of silver, or of tin, or of both; as to whether the salts of mercury exert some protective power, or merely kill the microbes. Dr. Hodgkin concludes that amalgam in a cavity near the gum, or in contact with enamel, lacks the elements of tooth-saving, and offers a "Norfolk oyster supper" for a case of amalgam fillings in two approximal cavities in molars or bicuspids, five years old, not now leaking, but still preserving the teeth, and the same reward for an amalgam filling against enamel, with the dentine gone, successfully preserving a tooth five years after insertion.

Adjourned.

